# Using Experimental Models to Decipher the Effects of Acetaminophen and NSAIDs on Reproductive Development and Health

**DOI:** 10.3389/ftox.2022.835360

**Published:** 2022-03-08

**Authors:** Brigitte Boizet-Bonhoure, Stéphanie Déjardin, Moïra Rossitto, Francis Poulat, Pascal Philibert

**Affiliations:** ^1^ Institute of Human Genetics, CNRS, University of Montpellier, Montpellier, France; ^2^ INRAE, University of Bordeaux, Bordeaux, France; ^3^ Laboratory of Biochemistry and Molecular Biology, Carèmeau Hospital, Nîmes University Hospital, Nîmes, France

**Keywords:** rodent models, fertility, ovary, testis, *in utero* exposure, NSAIDs, acetaminophen

## Abstract

Nonsteroidal anti-inflammatory drugs (NSAIDs), such as aspirin (acetylsalicylic acid), diclofenac and ibuprofen (IBU), and analgesic drugs, such as acetaminophen (APAP, or paracetamol), are widely used to treat inflammation and pain. APAP and IBU are over-the-counter drugs and are among the most commonly taken drugs in the first trimester of pregnancy, even in combination. Furthermore, these drugs and their metabolites are released in the environment, and can be frequently detected in wastewater, surface water, and importantly in drinking water. Although their environmental concentrations are much lower than the therapeutics doses, this suggests an uncontrolled low-dose exposure of the general population, including pregnant women and young children, two particularly at risk populations. Epidemiological studies show that exposure to these molecules in the first and second trimester of gestation can favor genital malformations in new-born boys. To investigate the cellular, molecular and mechanistic effects of exposure to these molecules, *ex vivo* studies with human or rodent gonadal explants and *in vivo* experiments in rodents have been performed in the past years. This review recapitulates recent data obtained in rodent models after *in utero* or postnatal exposure to these drugs. The first part of this review discusses the mechanisms by which NSAIDs and analgesics may impair gonadal development and maturation, puberty development, sex hormone production, maturation and function of adult organs, and ultimately fertility in the exposed animals and their offspring. Like other endocrine disruptors, NSAIDs and APAP interfere with endocrine gland function and may have inter/transgenerational adverse effects. Particularly, they may target germ cells, resulting in reduced quality of male and female gametes, and decreased fertility of exposed individuals and their descendants. Then, this review discusses the effects of exposure to a single drug (APAP, aspirin, or IBU) or to combinations of drugs during early embryogenesis, and the consequences on postnatal gonadal development and adult reproductive health. Altogether, these data may increase medical and public awareness about these reproductive health concerns, particularly in women of childbearing age, pregnant women, and parents of young children.

## Nonsteroidal Anti-Inflammatory Drugs and Analgesics: Use and Undesired Effects

### NSAID and Analgesic Exposure in Humans

NSAIDs, such as aspirin (acetylsalicylic acid, ASA), diclofenac (DCF) and ibuprofen (IBU), and analgesic drugs, such as acetaminophen (APAP, or paracetamol), are widely used to treat inflammation and pain (Kristensen et al., 2016). Their regular use by the general population is considered safe, and self-medication with analgesics is widespread. APAP and IBU are also among the most commonly prescribed drugs in the first trimester of pregnancy (Werler et al., 2005; McKenna and Mcintyre, 2006; Cejvanovic et al., 2014), and about 4% of pregnant women use APAP and NSAIDs in combination (Lind et al., 2017). In a puberty cohort study in Denmark, 54% of the mothers took APAP at least once during gestation (Ernst et al., 2019). Moreover, in a large cohort of 150,000 pregnant women, 29% of them took one over-the-counter NSAID (IBU, ASA, DCF, or naproxen) or one analgesic (APAP) during the first trimester, and more than 18% of them used APAP alone (Zafeiri et al., 2021). However, both APAP and NSAIDs can cross the placental barrier (Naga Rani et al., 1989; Siu et al., 2000) and are present in breast milk. In addition, APAP has been detected in human urine samples (Modick et al., 2014), and APAP and p-aminophenol (its primary metabolite) in male and female urine samples from 501 couples (Smarr et al., 2017). These data strongly suggest an environmental exposure besides the self-medication-related exposure. In agreement, IBU, DCF, and APAP have been detected in 2.5–23% of raw water and in 1–4% of treated water (low ng/L) samples (ANSES Laboratory, 2011) (Wee and Aris, 2017) and also in effluents from treatment plants and surface waters (few hundred ng/L) (Aydin et al., 2019; De Santiago-Martin et al., 2020; Palma et al., 2020), indicating that they are not completely removed at wastewater treatment plants. IBU and DCF are also often found in drinking water (Rabiet et al., 2006; Mompelat et al., 2009; Wang et al., 2017; Nantaba et al., 2020; Palma et al., 2020). Similarly, 2-hydroxyibuprofen, the main IBU metabolite, has been detected in more than 5% of raw and treated water samples (∼100 ng/L) (Han and Lee, 2017). This suggests an uncontrolled exposure of the general population, including pregnant women and young children, two particularly at risk populations.

### NSAID and APAP Exposure and Congenital Defects in Humans

Epidemiological studies found that exposure to APAP alone (Berkowitz and Lapinski, 1996) or in combination with NSAIDs during the first and second trimester of pregnancy is associated with higher cryptorchidism rate [for review (Kristensen et al., 2016; Stukenborg et al., 2021)]. Exposure to APAP, but not to IBU or ASA, for more than 4 weeks during the first and second trimester, has been associated with increased cryptorchidism occurrence in a cohort of 47,400 male newborns (Jensen et al., 2010). Similarly, the use of mild analgesics during the second trimester of pregnancy has been associated with congenital cryptorchidism, in a dose-dependent manner, in a birth cohort of 491 mothers (Kristensen et al., 2011a; Philippat et al., 2011). Also, in 3,184 women, APAP intake in the second trimester of pregnancy [14–22 gestational weeks (GW)], but not during the periconception period and the first trimester of pregnancy, increased the risk of congenital cryptorchidism, but not of hypospadias (Snijder et al., 2012). On the other hand, hypospadias incidence was significantly increased in 29,078 exposed newborns after NSAID intake by the mother in the last month before pregnancy and during the first trimester (Interrante et al., 2017).

More recently, in a large cohort of pregnant women, the consumption of at least one over-the-counter NSAIDs (IBU, ASA, DCF and/or naproxen) or analgesic (APAP) during the first trimester of pregnancy was significantly associated with higher risk of neonatal or perinatal health negative outcomes, such as premature delivery, neonatal death, low or high birthweight, neural tube defects, and hypospadias. The highest risk was conferred by the NSAID-APAP combination (Zafeiri et al., 2021). Conversely, APAP exposure during the third trimester was not associated with an increased risk of preterm birth and low birth weight (Castro et al., 2021). Similarly, APAP exposure from 4 weeks before pregnancy until delivery and during pubertal development has not been associated with male puberty disorders (based on the examination of secondary genital organs, pubic hair, adult voice, first ejaculation) in more than 7,000 boys. Conversely, in exposed girls (n > 7,800), earlier puberty was observed (based on breast development, pubic hair, acne, menarche) (Ernst et al., 2019). In a cohort of 259 premenopausal women, 69% reported use of over-the-counter analgesics. Moreover, intake of analgesics during the follicular phase of the menstrual cycle led to higher luteal progesterone secretion but did not affect gonadotropin and estradiol secretion (Matyas et al., 2015). On the basis of the many data on the adverse neurological and reproductive health effects associated with APAP maternal and perinatal intake, a consensus statement was recently released stressing the need of caution when using APAP during pregnancy (Bauer et al., 2021).

On the other hand, some epidemiology studies reported that *in utero* exposure to mild analgesics does not have any effect on reproductive disorders in boys and men (Stukenborg et al., 2021). No significant higher risk of major birth defects, spontaneous abortions or congenital defects was observed after exposure to IBU in the first trimester (German national Embryotox cohort: n > 1,000 exposed women) (Dathe et al., 2018). Similarly, APAP intake during the first trimester of pregnancy did not increase the risk of major congenital birth defects in 5,440 (Feldkamp et al., 2010) and 88,842 women (Rebordosa et al., 2008). Also, in a cohort of 6,246 boys, cryptorchidism incidence was not significantly increased by the mother’s use of drugs, such as APAP or ASA, during pregnancy (Wagner-Mahler et al., 2011). These heterogeneous results in epidemiological studies might be related to the difficulty to determine the exact administration timing and dose of NSAIDs/analgesics (Antonucci et al., 2012) during interviews that are often carried several months after the intake of such drugs. Moreover, within a study, the different effects observed after intake of different NSAIDs/analgesic molecules might also partly explained by their different specificity and their additional physiological targets (Dwivedi et al., 2015). Moreover, for a given NSAID molecule taken at the same time of gestation, the discrepancies on the occurrence of adverse events concerning the reproductive system may be explained by its intake concomitantly with other non-specific drugs that might modify its intrinsic effects. The fetal and neonatal adverse effects of NSAIDs can also be modulated by the presence of endocrine disruptors in the pregnant women’s environment or by their lifestyle (e.g., diet, use of cosmetics). Indeed, Wagner-Mahler et al. found that cryptorchidism detection at birth was associated with exposure to other endocrine disruptors, such as polychlorinated biphenyls and 1,1-dichloro-2,2-bis (pchlorophenyl) ethylene (Wagner-Mahler et al., 2011). Exposure to environmental endocrine disruptors has been associated with the occurrence of hypospadias and micropenis in male neonates (Raghavan et al., 2018; Gaspari et al., 2021) and with puberty disorders in girls (Sultan et al., 2018). Epidemiology studies on cryptorchidism occurrence in France in the last 12 years (*n* = 89,000 boys undergoing surgery for cryptorchidism), revealed an increase in its incidence by 36% that may be linked to geographically determined environmental factors (Le Moal et al., 2021).

### NSAIDs and Analgesics Are Endocrine Disruptors

Reproductive disorders, such as cryptorchidism, hypospadias and micro-penis, are the result of impaired androgen production by the fetal testis during the critical male programming window. This window was first defined in the rat (15.5–18.5 days post-coitum, dpc) (Welsh et al., 2008), and in humans should correspond to 8–14 GW (Welsh et al., 2008; Dean and Sharpe, 2013). The anogenital distance (AGD) is an indicator of the level of androgen exposure during the male programming window (Skakkebaek et al., 2001; Welsh et al., 2008; Dean and Sharpe, 2013; Fischer et al., 2020; Sharpe, 2020). Therefore, AGD measurement is used in epidemiological studies to evaluate the impact of drugs on the occurrence of genital disorders in newborn boys. Prenatal exposure to APAP during the human masculinization programming window (8–14 GW), but not before 8 GW or after 14 GW, has been associated with shorter AGD in male newborns, suggesting disruption of androgen action; however, shorter AGD was not associated with penis length and testis descent defects (Fisher et al., 2016). Furthermore, in a large population-based mother-child cohort, concomitant use by the mother of APAP and NSAIDs, but not of APAP alone, during pregnancy has been associated with shorter AGD in the male offspring. Conversely, no effect on AGD in girls was observed (Lind et al., 2017). In girls, little is known about AGD and its potential relation with the reproductive system (Mendiola et al., 2012). Nevertheless, AGD in newborn girls has been negatively associated with prenatal exposure to endocrine disruptors (such as phthalates) (Huang et al., 2009).

Thus, NSAIDs and APAP, like many environmental chemicals, might inhibit fetal testosterone production (Van Den Driesche et al., 2015; Kilcoyne and Mitchell, 2019; Sharpe, 2020; Stukenborg et al., 2021), thus acting as endocrine disruptors. Endocrine disruptors are molecules that interfere with the synthesis, transport, metabolism, or action of endogenous hormones, like many other endocrine disrupting compounds, such as phthalates, pesticides and phyto/xenoestrogens. The cumulative effects of NSAIDs and APAP and of other endocrine disruptors and the possibility of inter/transgenerational effects raise concerns about the long-term reproductive health of the general population. The conclusions of epidemiological studies on reproductive birth defects on reduced AGD occurrence following the use of NSAIDs or analgesics are heterogenous, and the exact timing, dosage and nature of the drugs that lead to congenital malformations have not been determined, yet. Thus, experimental models, *in vitro/ex vivo* rodent/human gonadal explants culture and *in vivo* studies in animal models have been developed to decipher the cellular and molecular impact of exposure to these drugs on reproductive health.

### NSAIDs and Analgesics Target Prostaglandins Production and Activity

#### NSAIDS and APAP Pharmacology

NSAIDs and APAP provide anti-inflammatory, anti-pyretic and analgesic effects to patients with various pathological conditions. Their anti-inflammatory effects are mediated through inhibition of cyclooxygenase 1 and 2 (COX-1 and COX-2) enzymatic activities that reduces vasodilatation and pain, induced mainly by prostaglandin (PG) E2 and prostacyclin (PGI2). The COX pathway includes the enzymes COX1-2 and COX-3, a splice variant of COX-1 in the central nervous system. This signaling cascade is involved in the conversion of arachidonic acid (AA) into PGH2 that is, then converted into various PGs (PGE2, PGD2, PGI2, PGF2α) by the action of specific terminal PG synthases (Cha et al., 2006; Korbecki et al., 2014). Specifically, AA is released from cell membrane phospholipids by phospholipase A2 (PLA2) and then can be converted into PGs through the COX pathway. It is also metabolized through two other enzymatic pathways, lipoxygenase (LOX) and cytochrome P450 (CYP450), leading to the production of leukotrienes and hydroxyeicosatetranoic acids (HETEs), respectively. The LOX pathway includes four enzymes (LOX-5, -8, -12 and -15) that catalyze the production mainly of leukotrienes (LTB4, LTC4, LTD4, and LTE4) which play various roles in inflammatory diseases and allergic disorders. Leukotriene and HETE metabolites modulate the secretion of pituitary hormones that regulate the ovary and testis physiology and potentiate the mechanism of action of luteinizing hormone in rat Leydig cell steroidogenesis through the upregulation of *StAR* gene expression (Cooke, 1999). In the CYP450 pathway, two enzymes (CYP450 epoxygenase and CYP450 ω-hydroxylase) catalyze the production of 20-HETE and eposyeicosatrienoic acids (EETs) that display anti-inflammatory effects by indirectly inhibiting COX-2 and 5-LOX expression (Hwang et al., 2013). Inhibition of the COX pathway by NSAIDs shunts the AA metabolism to the LOX pathway. This leads to excess production of leukotriene molecules, and suggest cross-talks between the pathways implicated in AA metabolism that might affect the responses to NSAIDs (Hwang et al., 2013).

NSAIDs and APAP have been classified on the basis of their chemical composition, mode of action, and COX specificity (Aronoff et al., 2006; Graham et al., 2013; Mazaleuskaya et al., 2015; Patrignani and Patrono, 2015; Lucas, 2016; Shah et al., 2016) (Table 1). APAP, IBU, naproxen, DCF, indomethacin and ASA are non-selective COX inhibitors, with some differences in the relative inhibition of COX-1 and COX-2 oxygenase active sites (Table 1). APAP also targets the COXs peroxidase (POX) active site and might target COX-3 in the central nervous system (Aronoff et al., 2006). Moreover, NSAIDs and APAP can recognize other physiological receptors and enzymes (e.g., PLA2, serum albumin, CYP450, prostaglandin ketoreductases, PPARγ), and this might contribute to their physiological effects (Dwivedi et al., 2015) (Table 1). Most NSAIDs (ASA, DCF) and APAP act as competitive inhibitors of PLA2, whereas indomethacin is categorized as a non-competitive inhibitor of PLA2. NSAID binding activity for these targets changes in function of their nature, although they all inhibit COX and PLA2 catalytic functions (Dwivedi et al., 2015). Furthermore, DCF, IBU, naproxen and indomethacin reduce leukotrienes production (5-HETE and LTC4) when used above the normal therapeutic doses (Gan, 2010). This suggests that these drugs can affect the different pathways of eicosanoid synthesis, depending on the cell type and the used dose.

**TABLE 1:** Pharmacology of NSAIDs and paracetamol.

	Chemical nomenclature	Cheminal structure	NSAIDs family	Action on COX	Half-life (hour)	COX specificity—IC50 (µM) in blood		Other target(s) (IC50)	Usual therapeutic concentration (µM)	Normal dose (mg/day)
						COX1	COX2			
Acetaminophen (APAP—paracetamol)	N-Acetyl-p-aminophenol or N-(4-hydroxyphényl)-acétamide	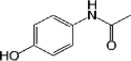	4-acetamido-phenol	Reversible non competitive inhibition	2	42.23	10.69	COX3 (ND)/PLA2(ND)/BRD2 (50 mM)	10–20	500–4000
Ibuprofen (High dose)	2-[4-(2-methylpropyl) phenyl]-propanoic acid	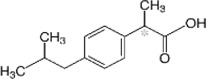	2-arylpropionate derivatives	Reversible competitive inhibition	1.8–3.5	5.9	9.9	HSA (KA = 1.0 × 10^5^M)/Lectoferrin (480 mM)/5-LOX (420 µM)	111	1200–2400
Ibuprofen (Low dose)									38.8	800–1200
Naproxen	2-(6-methoxynaphthalen-2-yl)-propanoate	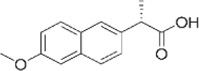	2-arylpropionate derivatives	Reversible competitive inhibition	12–17	32.01	28.19		1.3	550–1100
Diclofenac	2-(2,6-dichloranilino) phenylacetic acid	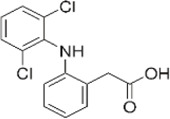	phenylacetic acid derivatives	Reversible competitive inhibition	1–2	0.26	0.01	PLA2 (48 nM)/CYP450 KD = 50 mM	6.1	150–200
Indomethacin	1- (p-chlorobenzoyl)25-methoxy-2-methylindole-3-acetic acid	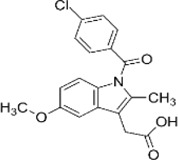	indolacetic acid derivatives	Time-reversible inhibition	2.6–11.2	0.21	0.37	PLA2 (KD = 1.3 mM)/HSA (KA = 2.5 × 10^5^M)/PTGR (2.1 mM)/PPARg (KD = 9.73 mM)/Lectoferrin KD = 260 mM	0.4–2.6	75–200
Aspirin (High dose)	2-acetoxybenzoic acid	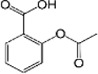	salicylates	Irreversible time-dependent inactivation	0.3 (3 h salicylic acid)	4.45	13.88	PLA2 KD = 6.4 mM/HSA KD = 70 mM	100–150	1200–5200
Aspirin (Low dose)									4.9–18.6	80–325

IC50 = concentration of drug required to inhibit the enzymatic activity. ND: Not determined. PLA2: phospholipase A2. HSA: Human serum albumin. PTGR: prostaglandin keto reductase. PPARg: peroxisome proliferator-activated receptor gamma.

Central inflammatory and nociceptive pathways also are potential NSAID targets. This central action involves COX enzyme upregulation in spinal cord that increases PGE2 production (Vuilleumier et al., 2018). NSAIDs and APAP interact with endocannabinoids, and the serotonergic, noradrenergic and cholinergic systems in the central nervous system, and this contributes to their analgesic effect (Hamza and Dionne, 2009). In the central nervous system, IBU, DCF, ASA and indomethacin influence the expression of factors involved in inflammatory responses (IL-6, MMPs, TIMPs) and APAP inhibits the membrane receptor that releases endocannabinoids from post-synaptic neurons (Hamza and Dionne, 2009). This is important because, together with steroids and cytokines, endocannabinoids that also originate from AA play important roles in the human reproduction (fertilization, pregnancy, and fetal development) (Battista et al., 2008).

#### Prostaglandins Are Involved in Reproduction and Are Targeted by NSAID and APAP

PG receptors and PG synthases (PGDS, PGES, and PGIS) are widely expressed in undifferentiated gonads (Malki et al., 2005), and PGD2 signaling is involved in Sertoli cell differentiation by increasing the expression of *Sox9* and then participating in the early steps of testis organogenesis [both somatic (Moniot et al., 2009; Moniot et al., 2011) and germline cells (Moniot et al., 2014)]. Male and female rat fetal gonads expressed COX2 and the PGE2 receptor *Ep2* (Dean et al., 2016). PGs are essential for male differentiation because in mouse fetuses, PG level modulation can inhibit testosterone synthesis and the development of male genitalia (Gupta and Goldman, 1986; Gupta, 1989).

PGs are produced in postnatal and adult (female and male) reproductive organs where they are implicated in many physiological (e.g., steroidogenesis) and pathological processes (Cha et al., 2006; Fortier et al., 2008; Frungieri et al., 2015; Rossitto et al., 2015; Urade, 2021). PG receptors and PG synthases have been detected in human Leydig cells (Tokugawa et al., 1998; Schell et al., 2007). Mice in which the gene encoding PGD2 synthase was ablated and thus PGD2 is not produced, display unilateral cryptorchidism (Philibert et al., 2013). These data suggest that PG secretion might be related to androgen production in testis. In humans, PGs are abundant in semen; however, it is thought that they are produced mainly in the seminal vesicles.

PGs are also involved in the female reproductive tract physiology (Kennedy et al., 2007): ovulation and fertilization (Kobayashi and Narumiya, 2002), different stages of blastocyst implantation (Salleh, 2014), induction of labor and parturition (Challis et al., 2002), and different endometrial pathologies (Sales and Jabbour, 2003; Philibert et al., 2021). PGE2 acts as a germ cell regulator during ovary development (Bayne et al., 2009).

These wide PG synthase and receptor expression and PG production suggest that NSAIDs and APAP, through COX inhibition, might affect the development and maturation of embryonic, postnatal and adult gonads and of the reproductive tract. Indeed, exposure of female rat fetal gonads to APAP or indomethacin significantly reduces PGE2 levels in 17.5 dpc exposed ovaries (Dean et al., 2016). Similarly, in mice, *in utero* exposure to therapeutic doses of the APAP-IBU combination during the sex determination period leads to decreased production of PGD2, PGE2, and PGI2 (as measured by its metabolite 6-keto-PGF1α) in 13.5 dpc APAP+ IBU exposed testes (Rossitto et al., 2019a) and ovaries (Rossitto et al., 2019b). Recent additional *ex vivo* and *in vivo* studies confirmed that NSAID and APAP treatment/exposure interferes with PGs (PGD2, PGE2) production, although the relationships between PGs production and steroidogenesis remain uncertain, as discussed in paragraphs 3 and 4 of this manuscript. Besides the inhibition of PG synthesis, other mechanisms, unrelated to COX inhibition, are probably involved in the induction of antiandrogenic effects and male genital abnormalities in animal models.

## Development and Maturation of the Reproductive Tract and Potential Risks of Disorders

### Development of Gonads and Reproductive Tract

In most mammals, in accordance with the egg karyotype at fertilization, the determination of somatic sex is initiated in undifferentiated embryonic gonads at 10.5–12.5 dpc in mice and at ∼week 7 of gestation in humans *via* the expression of male (*SRY*, *SOX9*) or female (*RSPO1*, *WNT4*) specific genes (Nef et al., 2019; Yildirim et al., 2020), and is actively maintained throughout reproductive life (Jimenez et al., 2021). Once differentiated, the somatic-supporting lineage (Sertoli cells in males and granulosa cells in females) controls the differentiation of germ cells, the precursors of gametes that are crucial for reproductive function. Germ cell sexual determination, i.e., mitotic arrest in males and onset of meiosis in females, is initiated between 12.5 and 15.5 dpc in mice (8–9 GW) (Spiller and Bowles, 2019).

Besides embryonic gonads, the genital tract includes two duct types (Wolffian and Müllerian ducts) that will give rise to the secondary sexual organs (Yildirim et al., 2020). Once formed, the embryonic testis through the differentiated Sertoli cells, produces anti-Müllerian hormone (AMH) that induces the Müllerian duct regression. From 12.5 dpc in mice (14.5 dpc in rats, and 7–8 GW in humans), fetal Leydig cells produce Insulin-like factor 3 (Insl3) and testosterone that regulate the first and second phase of testis descent, respectively. Testosterone causes the urogenital tract virilization in two ways. First, it induces the differentiation of Wolffian ducts into vas deferens, seminal vesicles, and epididymis. Second, in the urogenital sinus and external genitalia, testosterone is rapidly and locally transformed into dihydrotestosterone that induces urethral and prostate development in boys, and also penis and scrotum formation (Makela et al., 2019). This androgen production during the masculinization programming window (between 15.5 and 18.5 dpc in the rat and 8–14 GW in humans) is critical for programming the male reproductive tract development (Sharpe, 2020). In females, Müllerian ducts will give rise to the reproductive tract (vagina, uterus, fallopian tubes).

Activation of the hypothalamic-pituitary-gonadal axis leads to the postnatal testis maturation (Meroni et al., 2019). When puberty begins, AMH expression decreases as a direct consequence of androgen action in Sertoli cells (Edelsztein and Rey, 2019; Meroni et al., 2019) that induces their maturation and the establishment of Sertoli-Sertoli and Sertoli-germ cell junctions involved in blood-testis-barrier formation. Sertoli cells provide the structure of seminiferous tubules (Sharpe et al., 2003; Franca et al., 2016; Sharpe, 2020) and the environment for the regulation of germ cells that will be engaged into the first wave of spermatogenesis at ∼ 8 days post partum (dpp) in mice (puberty in humans) after meiosis initiation (Makela et al., 2019). Throughout the adult reproductive life, spermatogonial stem cells will undergo spermatogenesis, that is, finely regulated by epigenetic regulators (Hammoud et al., 2015; Tseng et al., 2015), and is directly related to fertility (Bissonnette et al., 2009). In response to luteinizing hormone, steroidogenesis is induced in fetal Leydig cells to produce androgens that are necessary during puberty and adulthood for the development of the male secondary sexual characteristics and for the initiation and maintenance of spermatogenesis (Zirkin and Papadopoulos, 2018). Testosterone participates in the development of adult Leydig cells that gradually replace fetal Leydig cells.

In female mice, at birth, oocytes arrested in the meiotic diplotene/dictyate stage individualize and become surrounded by granulosa cells to form primordial follicles (Pepling, 2012). At the same time, many germ cells undergo apoptosis up to 4 dpp (Wear et al., 2016) when the final stock of oocytes in mouse ovaries is established. This primordial follicle pool size will largely determine the female reproductive lifespan (Mork et al., 2012 9446; Pepling, 2012). Gametogenesis begins with the first wave of folliculogenesis to produce ovulatory follicles at each ovarian cycle in mature animals (Jagarlamudi and Rajkovic, 2012). The oocyte blocked in prophase I of meiosis gradually acquires its definitive epigenetic marks (DNA methylation and genetic fingerprinting), then enters metaphase I at the time of ovulation, and completes its maturation (metaphase II) only after fertilization (Suzuki et al., 2015; Puttabyatappa and Padmanabhan, 2018). Dysregulation of the signaling cascades that orchestrate cell proliferation and cell death and that determine whether a follicle will continue to develop or undergo atresia can disturb the balance between primordial follicle dormancy and activation (Huhtaniemi et al., 2018).

### Adult Reproductive Health and Reproductive Tract Disorders

Reproductive health defines adult fertility and more broadly the reproductive processes and functions throughout life. It also includes reproductive tract pathology, such as cancer, urogenital malformations and inter/transgenerational reproductive effects, and can be monitored using the following specific indicators (Le Moal et al., 2016):

- in men: sperm quality (concentration, mobility, morphological quality), the incidence of urogenital malformations (cryptorchidism, hypospadias) and of testis or prostate cancer;
- in women: the ovarian reserve of oocytes (AMH quantification), the incidence of early puberty, of polycystic ovary syndrome, of early ovarian failure (early menopause), and of endometriosis.

Many studies have highlighted reproductive function changes in humans. Genital malformations in newborn boys, such as cryptorchidism and hypospadias, have gradually increased in the last 3 decades. These disorders are symptoms of a single entity that is, defined as Testicular Dysgenesis Syndrome (TDS), and that may be caused by genetic abnormalities (Skakkebaek et al., 2001). In TDS, the decreased function of Sertoli and Leydig cells affects androgen production and germ cell development, resulting in increased infertility and higher risk of testicular cancer in adult men (Skakkebaek, 2016). More recently, ovarian dysgenesis syndrome also has been described (Johansson et al., 2017; Puttabyatappa and Padmanabhan, 2018).

The etiology of these syndromes is not fully known, but the steady increase in reproductive disorders suggests causes related to lifestyle and/or environmental factors rather than genetic alterations (Johansson et al., 2017; Arendrup et al., 2018). Many epidemiological and animal studies showed that *in utero* exposure to endocrine disruptors interferes with endocrine gland functions that have been implicated in hypospadias and micropenis development in male neonates (Skakkebaek, 2016; Van Den Driesche et al., 2017; Raghavan et al., 2018; Gaspari et al., 2021). Environmental factors acting during prenatal or adult life are involved in early puberty (Sultan et al., 2018) and ovarian reserve decrease (Arendrup et al., 2018), and could contribute to premature ovarian insufficiency (Hoyer and Keating, 2014; Vabre et al., 2017; Huhtaniemi et al., 2018). Furthermore, the critical window of exposure overlaps with the fetal gonadal sex determination period that coincides with the period of epigenetic reprogramming of developing germ cells (Skakkebaek, 2016). Thus, endocrine disruptors may have inter/transgenerational adverse effects, particularly in germ cells (Wei et al., 2015), resulting in reduced quality of gametes, sperm and oocytes, and fertility.

## Effects of Exposure to NSAIDs and APAP *in Vitro/ex Vivo* Studies (Rodent and Human Gonadal/Cells Tissues)

To understand the heterogenous results of epidemiological studies on the impact of mild analgesics on gonad development and function, *ex vivo* studies in rodents and *in vitro*/*ex vivo* studies using human cells or testis explants have been performed in the last decade. These experiments were carried out using drug concentrations in the range of the expected plasmatic levels at 30 min to 1 h after administration of therapeutic doses in humans (Table 1). In *in vitro* and *ex vivo* studies, the equivalent of APAP, ASA, IBU and indomethacin therapeutic doses ranged from 10^−4^ to 10^−6^ M, which corresponded to plasmatic concentrations of 1.37 × 10^−4^ M for APAP (Rayburn et al., 1986), 2 × 10^−4^ M for ASA (Needs and Brooks, 1985), 1.45 × 10^−4^ M for IBU (Rainsford, 2009) and 1.5 × 10^−6^ M for indomethacin (Ben Maamar et al., 2017), in humans.

### *Ex Vivo* Culture of Rodent Fetal Gonads

In *ex vivo* organotypic cultures of 14.5 dpc rat testes, testosterone secretion was significantly reduced by 50% upon exposure to 10^−6^ M APAP only after a 48 h or 72 h of culture or to 10^−5^ M ASA throughout the assay period (24–72 h). However, PGD2 production was decreased by 30% only after exposure to 10^−6^ M APAP for 24 h or to 10^−5^ M ASA for 48–72 h, suggesting no correlation between the drug inhibitory effects on PGD2 production and on testosterone production (Kristensen et al., 2011a). After exposure of 14.5 dpc rat testes to APAP (10^−7^ M–10^−4^ M), ASA (10^−6^ M–10^−4^ M) or indomethacin (10^−6^ M–10^−5^ M) for 3 days, testosterone production was significantly decreased at the highest doses (i.e., their therapeutic plasmatic concentration) (Kristensen et al., 2012). However, Leydig cell number was not affected, and APAP did not decrease INSL3 production. Exposure to 10^−6^ M and 10^−5^ M indomethacin, but not to APAP or ASA, significantly decreased PGD2 levels (Kristensen et al., 2012). Thus, both studies showed that APAP, ASA and indomethacin have anti-androgenic effects at 10^−5^ M in 14.5 dpc rat testes. The PGD2 production was not correlated with testosterone secretion, suggesting that their anti-androgenic effect occurs through a COX-independent mechanism. Alternatively, this difference could be explained by the fact that testosterone and PGD2 are secreted by different cell types in testes [i.e., Sertoli cells for PGD2 (Moniot et al., 2009), and Leydig cells for testosterone] or by the different targets of these drugs (APAP: COX-2, ASA: COX-1, and indomethacin: COX-1 and COX-2, Table 1).

The hypothesis that PGD2 production by embryonic Sertoli cells might be affected was confirmed by the use of the SC5 juvenile mouse Sertoli cell line cultured in the presence of NSAIDs (IBU, ASA, indomethacin) or APAP, at concentrations between 10^−7^ and 10^−5^ M, for 24 h. A dose-dependent reduction in PGD2 production was observed, similarly to what obtained with endocrine disruptors, such as bisphenol A, diethylstilbestrol and phthalates. A similar reduction in PGE2 production in SC5 cells was observed after incubation with dibutyl phthalate, n-butylparaben or bisphenol A, suggesting that these endocrine disruptors inhibit the upstream enzymes of the PG synthesis pathway (i.e., COX-1 and COX-2) because their effect did not implicate the PGD2 and PGE2 receptors and the nuclear peroxisome proliferator-activated receptors (Kristensen et al., 2011b).

### *Ex Vivo* Culture of Human Fetal and Adult Gonadal Explants and Culture of Human Cell Lines

Organotypic culture of human fetal testis explants (7–12 GW) in the presence of APAP, ASA or indomethacin (at doses 10^−5^ M, equivalent to therapeutic doses) showed heterogeneous effects on testosterone, INSL3 and AMH production. Indomethacin and ASA induced testosterone production in 8–9 GW testis explants cultured for 72 h, whereas APAP decreased INSL3 levels, and ASA activated AMH production (Mazaud-Guittot et al., 2013). However, in similar cultures of 10–12 GW human fetal testes, incubation with APAP, IBU, ASA or indomethacin for 24 h did not significantly affect testosterone production (Gaudriault et al., 2017), suggesting an age window of sensitivity to these drugs. Exposure to IBU also affected Sertoli cell function in human fetal testes (7–8 GW and 8–12 GW), as indicated by the reduced AMH production compared with untreated controls, and the decreased expression of *AMH* and *SOX9* (Sertoli cell differentiation markers). This implies that the endocrine function of human fetal Sertoli cells is affected not only by ASA (induction), but also by IBU (inhibitory effect) (Mazaud-Guittot et al., 2013; Ben Maamar et al., 2017). Similarly, exposure of human fetal testes (7–17 GW) to IBU (10^−7^–10^−4^ M) suppressed in a dose-dependent manner testosterone and INSL3 only in 8–9 GW fetal testes, by reducing the expression of some steroidogenic genes (*CYP11A1*, *CYP17A1*, *HSD17B3*) (Ben Maamar et al., 2017). Therefore, major differences in the responses to APAP, ASA, indomethacin and IBU in terms of hormone production (testosterone, INSL3, AMH) were observed in fetal human testes exposed during the male programming window.

The significant inhibition of PGE2 but not of PGD2 production by APAP, ASA, indomethacin (Mazaud-Guittot et al., 2013) and IBU (Ben Maamar et al., 2017) only in 7–12 GW testes suggests a critical window for PG sensitivity. However, IBU (10^−5^ M) suppresses ovarian PGE2 production at all developmental stages (Leverrier-Penna et al., 2018). PGD2 production seems to be less affected than PGE2 production by exposure to NSAIDs and APAP (Mazaud-Guittot et al., 2013; Ben Maamar et al., 2017). This is different from the findings obtained in *ex vivo* organotypic cultures of 14.5 dpc rat testes in the presence of APAP and ASA (Kristensen et al., 2012), suggesting that the mechanism of PG synthesis inhibition by these drugs might depend on the species and/or on the different culture settings.

These various physiological effects of NSAIDs and APAP on the fetal testis endocrine functions might be the consequence also of additional interactions of these drugs with other physiological receptor and enzymes, such as PLA2, serum albumin (HAS), CYP450, prostaglandin ketoreductase (PTGR), PPARγ (Dwivedi et al., 2015) (Table 1). Furthermore, inhibition of the COX pathway by NSAIDs shunts the AA metabolism to the LOX pathway, leading to excess production of leukotriene molecules (Hwang et al., 2013). Thus, leukotriene and HETE metabolites, which modulate the secretion of pituitary hormones and potentiate luteinizing hormone mechanism of action in rat Leydig cell steroidogenesis, might also be implicated in NSAID and APAP effect in fetal testis (Cooke, 1999).

Exposure to these drugs (APAP, ASA, or indomethacin) does not modify testis histology, the number of germ cells, and the germ cell/Sertoli cell ratio in 8–12 GW human testis explants (Mazaud-Guittot et al., 2013). However, addition of therapeutic doses of APAP (10^−5^ M) and IBU (10^−5^ M) to hanging drop cultures of human fetal testes and ovaries (8–11 GW) reduced the total number of germ cells as well as the number of KI-67 positive gonocytes, and increased the number of apoptotic cells (Hurtado-Gonzalez et al., 2018). Similarly, exposure of *ex vivo* organotypic cultures of human fetal ovaries (7–12 GW) to IBU (10^−6^–10^−4^ M) led to a decrease in the germ cell pool due to a reduction in their proliferation through upregulation of the cell cycle inhibitors *TP53* and *CDKNIA* (p21) and apoptosis increase. The significantly reduced expression of the specific germ cell markers *TPAP2C*, *LIN28A* and *KIT* suggests that also germ cell growth and differentiation are altered upon IBU exposure (Ben Maamar et al., 2017). The differential effects of the exposure to these drugs on germ cells might not be due to the drug specific inhibition of COX-1 or COX-2 (APAP, COX-2; ASA and IBU, COX-1); however, the discrepancy concerning APAP effect in germ cells might be related to the different culture types used in these studies. The larger range of IBU effects on all testis cell types might be explained by additional targets of IBU in the human fetal testis (e.g., the LOX pathway).

Similar experiments performed using adult testis explants showed that exposure to APAP, ASA or indomethacin (10^−5^–10^−4^ M) for 24 h significantly decreases testosterone secretion. On the other hand, INSL3 production was affected only by ASA and indomethacin, and ASA altered inhibin B production. PGD2 and PGE2 secretion was influenced by all these drugs (Albert et al., 2013). These data suggest that adult Leydig cells might be more sensitive to these drugs than fetal Leydig cells. In adult men, IBU disrupts the hypothalamic-pituitary-gonadal axis, thus leading to increased luteinizing hormone secretion and decreased testosterone production. *Ex vivo* studies using adult testis explants confirmed IBU anti-androgenic action (Kristensen et al., 2018). Moreover, APAP and its metabolite *p*-aminophenol (quantified in urine) have been associated with reduced sperm quality in 500 male partners of couples planning for pregnancy (Smarr et al., 2017).

Steroidogenesis monitoring in the NCI-H295R human adrenocortical carcinoma cell line incubated with APAP (from 10^−7^ to 10^−3^ M) and aniline (from 10^−10^ to 10^−3^ M), a central APAP precursor, showed that aniline increases the levels of progesterone and 17α-hydroxyprogesterone and also of testosterone (Holm et al., 2015). In the same cell line, the analgesic dipyrone (metamizole) and its main metabolites induced similar changes in steroid hormone production, including decreased testosterone and increased 17-OH progesterone concentrations (Passoni et al., 2018). Conversely, APAP increased pregnenolone, but impaired its subsequent conversion to progesterone by CYP17A1 (Holm et al., 2015). APAP also modulated the expression of steroidogenic genes, leading to a significant dose-dependent decrease in estradiol secretion in human placental JEG-3 cells (Addo et al., 2021). Furthermore, the anti-androgenic and anti-estrogenic activities of anti-inflammatory drugs (APAP, IBU, DCF, naproxen) at doses between 10^−6^ and 10^−5^ M have been demonstrated using two *in vitro* reporter-gene assays based on a CXCL12-expressing human T47 breast carcinoma cell line and ERα and AR-expressing recombinant yeast assays (Ezechias et al., 2016). Incubation of the NTera2 human germ cell tumor–derived cell line with APAP (10^−5^ M or 5 × 10^−5^ M) and IBU (10^−5^ M), or antagonists of the PGE2 receptors EP2 and EP4 decreased cell proliferation and increased cell death. Addition of EP2+EP4 agonists to APAP-treated NTera2 cells normalized the cell count, indicating that APAP-induced NTera2 cell loss requires an intact PGE2 signaling pathway. In addition, expression of the gonocyte pluripotency marker *OCT4* and of the DNA methyltransferases *DNMT3a* and *DNMT3b* was significantly downregulated in treated compared with control cells (Hurtado-Gonzalez et al., 2018). APAP also affected the expression of angiogenesis and vascular remodeling genes in HTR-8/SVneo cells (derived from first trimester human trophoblasts) that express the COX-1 and COX-2 cyclooxygenases (Burman et al., 2021).

Thus, analgesics administered at doses equivalent to the plasmatic concentrations in humans behave as endocrine disruptors depending on the stages of the urogenital tract development and on treatment duration*.* Exposure to therapeutic concentrations of APAP, IBU, and ASA during the specific “windows of sensitivity” has multiple effects (development and function) on Sertoli, Leydig and germ cells in *ex vivo/in vitro* experiments, including on the production of sexual hormones and PGs. However, *ex vivo* culture of rat or human gonadal explants showed no clear link between testosterone deficiency induced by NSAIDs/analgesics and PG production inhibition. Moreover, it is not clear whether PGE2 and/or PGD2 are involved in these processes. Additional studies are needed to elucidate whether other AA metabolites are implicated in the effects observed after exposure to NSAIDs, and whether other NSAIDs targets are involved in the hormonal effects in gonads. However, the effect on germ cell proliferation suggests that APAP or IBU intake by pregnant women during the early stages of gonadal organogenesis might impair the establishment of the stem cell pool in adult organs with negative consequence on fertility in adult life.

## *In vivo* Experiments in Rodents to Elucidate the Impacts of NSAID and APAP Undesired Effects on Reproductive Health (Table 2)

To address these questions, *in vivo* rodent models have been developed to evaluate the gonadotoxic effects and the mechanisms of action of these drugs, and to consider also their transformation into metabolites, the central endocrine regulations, and the gonad microenvironment. To set up these experiments, the dose-equivalents between humans and rat/mice must be calculated by taking into account the body weight of an individual (reference for humans: 60 kg and for a CD1 mice: 30 g) and the specific pharmacokinetic features of each species (Davidson et al., 1986). Particularly, pharmacokinetics in mice and rats is 10 times higher than in humans (Davidson et al., 1986). Thus, as in humans, the recommended therapeutic doses of APAP and IBU are 20–50 mg/kg/day (1–3 g/day) and 10 mg/kg/day (0.6 g/day), the equivalent “therapeutic” doses used in rodents, were increased to 60–150 mg/kg/day and 15–30 mg/kg/day, respectively.

### Male Reproductive Health

Prenatal exposure of pregnant rats (13–21 dpc) to APAP (150–350 mg/kg/day) resulted in reduced AGD in the 21 dpc exposed offsprings at all doses. However, fetal testis testosterone production was not significantly modified, whereas it was decreased in *ex vivo* culture of fetal testes at the male programming window in the presence of equivalent APAP doses (Kristensen et al., 2011a). The litters of pregnant mice treated with APAP (50 or 150 mg/kg/day) or aniline (31 or 93 mg/kg/day) by gavage between 7 and 20 dpc were monitored after birth and in adulthood (Holm et al., 2015). Litter size, sex ratio and pup weight were similar in exposed and control litters. AGD decrease was dose- and age-dependent. However, analysis of exposed adult testes did not highlight significant defects in germ cell and Leydig cell number (Holm et al., 2015). This suggests that *in vivo*, APAP might be metabolized and was not associated with testosterone secretion inhibition. Indomethacin (0.8 mg/kg/day) exposure of pregnant rats during the masculinization window (15.5–18.5 dpc) led to decreased fetal body and testis weight at 21.5 dpc and reduced testis PGE2 production, without any effect on testosterone secretion (Dean et al., 2013) (Table 2). In agreement with the *ex vivo* experiments [see *Effects of Exposure to NSAIDs and APAP in Vitro/ex Vivo Studies (Rodent and Human Gonadal/Cells Tissues*)], this confirmed that androgen secretion and PGs production are not linked and that indomethacin might affect different metabolic pathways that compensate the primary COX-mediated inhibitory effects. *In utero* exposure (15–21 dpc) of rats to IBU (10–60 mg/kg/day) can affect the hypothalamus programming in the male offspring. In males exposed to IBU, body weight and AGD were decreased, and testis descent and preputial separation (puberty onset) were delayed. In adult age, serum testosterone levels and the percentage of sperm with normal morphology were decreased, but fertility and male behavior were normal (Balin et al., 2020). Exposure of female rats to APAP (350 mg/kg/day during gestation and lactation) did not affect the AGD and preputial separation (at 21 dpp) and testosterone production and sperm parameters (in adulthood) in their male offspring. However, sexual behavior (first and number of ejaculations) was significantly reduced in the exposed male offspring, suggesting impaired sexual hypothalamic differentiation (Pereira et al., 2020). These effects on puberty onset and sexual behavior and the differential effects between *ex vivo* and *in vivo* studies, might be the consequences of the central effects of IBU and APAP, in addition to their peripheral effects.

**TABLE 2:** *In vivo* studies in rodents on effects of mild analgesics on gonadal development and function.

Species	Method	Exposure period	Compound	Doses	Effects	Reference
Rat	*in vivo*	13–21 dpc	APAP	150–250–350 mg/kg/day	Reduced AGD in 21 dpc exposed offsprings for both doses no effect on testosterone production	Kristensen et al. (2011a)
Rat	*in vivo*	15.5–18.5 dpc	Indomethacin	0.8 mg/kg/day	decreased fœtal body, testis weight at 21.5 dpc and testicular PGE2 production but no effect on testosterone secretion; no effect on postnatal and adult animals	Dean et al. (2013)
Mouse	*in vivo*	7–20 dpc	APAP	50 or 150 mg/kg/day	reduced AGD dependently on the doses and on the age (4–10-week-old)	Holm et al. (2015)
			Aniline	31 or 93 mg/kg/day	reduced AGD dependently on the doses and on the age (4–10-week-old)	
Rat	*In vivo*	15 dpc to 21 dpp	IBU	10–60 mg/kg/day	decreased in body weight and AGD; delayed testicular descent and preputial separation reduced serum testosterone levels and normal sperm morphology but normal fertility and male behavior	Balin et al. (2020)
Rat	*in vivo*	6 dpc–21 dpp	APAP	350 mg/kg/day	reduced sexual behavior (first and number of ejaculations) in male exposed offspring Normal AGD, preputial separation (21 dpp) or testosterone production and sperm parameters in adults	Pereira et al. (2020)
Rat	*in vivo*	13.5 dpc	APAP	350 mg/kg for 1 day	decreased testosterone production in 17.5 dpc testes reduced AGD in 21.5 dpc exposed fœtus, through the reduced expression of steroidogenic enzymes (Cyp11a1 and Cyp17a1)	van den Driesche et al. (2015)
Mouse	Human Testis xenograft	14–20 GW	APAP	20 mg/kg, 7 days (3 times daily) or 350 mg/kg 7 days (1 time daily)	decreased plasma testosterone levels and seminal vesicle weight	
				20 mg/kg, 1 day (3 times daily) or 350 mg/kg for 1 day	no effect on testosterone production	
Mouse	Human Testis xenograft	14–17 GW	IBU	10 mg/kg, 7 days (3 times daily)	no effect on testosterone or AMH production	Ben Maamar et al. (2017)
Mouse	Human Testis xenograft	14–17 GW	APAP	20 mg/kg, 7 days 7 days (3 times daily)	reduced total germ cell number by 43%	Hurtado-Gonzalez et al. (2018)
			IBU	10 mg/kg/day 7 days (3 times daily)	reduced total germ cell number by 53%	
Rat	*in vivo*	13.5–21.5 dpc	APAP	350 mg/kg/day	earlier gonocyte differentiation no effect on F1 male fertility	Dean et al. (2016)
		15.5–18.5 dpc	indomethacin	0.8 mg/kg/day	reduced germ cells number in fœtal testis earlier gonocyte differentiation no effect on F1 male fertility	
		13.5–21.5 dpc	APAP	350 mg/kg/day	decreased PGE2 production in 17.5 dpc ovary and reduced germ cell number; decreased fertility of F1 females reduced (by 60%) primordial follicle numbers in F2 offspring and increased in serum AMH levels in F2 offspring	
		15.5–18.5 dpc	indomethacin	0.8 mg/kg/day		
Mouse	*in vivo*	10.5–13.5 dpc	APAP and IBU (combination)	50 mg/kg/day and 15 mg/kg/day	decreased embryonic germ cell proliferation reduced spermatogonia A pool and delayed Sertoli cell maturation reduced testosterone production by 80% and reduced sperm concentration in adult F1 animals; reduced sperm mobility and subfertility of F2 animals	Rossitto et al. (2019a)
			APAP and IBU (combination)	50 mg/kg/day and 15 mg/kg/day	Increase number of female germ cells at 13.5 dpc delay in the onset of meiosis decrease in follicular stock in F1/F2 impaired regression of corpora lutaea	Rossitto et al. (2019b)
Mouse	*in vivo*	7 dpc to delivery	APAP	50 or 150 mg/kg/day	decreased number of 13.5dpc primordial germ cells leading to a reduced follicular pool; no effect on male PGCs decreased female fertility after 6-months (numbers of full-term pregnancies and number of pups per litter)	Holm et al. (2016)
Mouse	*in vivo*	5–18.5 dpc	ASA IBU	14.3 mg/kg/day 5.6 mg/kg/day	no effect on adult mouse sperm parameters	Stutz et al. (2000)
Rat	*in vivo*	6 dpc–21 dpp or 6 dpc to end of lactation	APAP	350 mg/kg/day	decreased follicle reserve and increased plasma oestradiol concentrations in female offspring impaired sexual behaviour	Aleixo et al. (2020)
Rat	*in vivo*	23–53 dpp	IBU	2.4–14.3 mg/kg/day	impaired sperm parameters: decreased sperm motility (60%) and daily sperm production (30%) reduction in testosterone secretion	Barbosa et al. (2020)
		23–53 dpp	IBU	2.4–14.3 mg/kg/day	impaired œstrous cyclicity reduced fertility potential	
Rat	*in vivo*	Adult: 30 consecutive days	APAP	500 or 1,000 mg/kg/day	reduced sexual competence, sperm count and quality, and fertility Reversible effects	Ratnasooriya and Jayakody (2000)
Rat	*in vivo*	13–16 dpc	APAP without 12 other endocrine-disrupting chemicals	360 mg/kg/day	decreased AGD and ventral prostate weight, increased nipple retention at 13 dpp	Axelstad (2014)
				360 mg/kg/day	increased 22 dpp testis weight and impaired testis histology decreased sperm count (17–23%) in adult	Axelstad (2018)
		7 dpc–22 dpp		360 mg/kg/day	reduced primordial follicle number and decreased expression of ovocyte markers; earlier puberty and irregular œstrus cycles	Johansson et al. (2016)
		7 dpc–22 dpp		0.8 mg/kg/day	delayed puberty onset, altered folliculogenesis in F2 and F3 animals but not in F1 transgenerational alteration of hypothalamic gene expression	López-Rodríguez (2021)

#### Human Fetal Testis Tissue Xenografts

*Ex vivo* cultures of human fetal testes have been validated in xenograft models in nude mice to mimic the human *in utero* environment (Van Den Driesche et al., 2015; Ben Maamar et al., 2017; Hurtado-Gonzalez et al., 2018) (Table 2). After human fetal testis tissue (14–20 GW) grafting, plasma testosterone levels and seminal vesicle weight in graft recipient mice were decreased following treatment with human-relevant doses of APAP (20 mg/kg three times per day) for 7 days. Conversely, treatment for only 1 day or with a single daily high dose of APAP (350 mg/kg/day) did not have any effect on testosterone production (Van Den Driesche et al., 2015). Treatment of pregnant rats with a comparable APAP high dose decreased testosterone production by 17.5 dpc testes and the AGD index in 21.5 dpc exposed fetuses, through downregulation of steroidogenic enzymes (*Cyp11a1* and *Cyp17a1*) in rat fetal testes (Van Den Driesche et al., 2015). On the other hand, exposure to IBU at clinical doses (10 mg/kg, 3 times per day, for 7 days) did not affect testosterone and AMH production by human fetal (14–17 GW) testis tissue grafted in mice (Ben Maamar et al., 2017). In mice xenografted with second-trimester testis tissue, treatment with APAP (20 mg/kg/day) or IBU (10 mg/kg/day) for 7 days (3 times per day) significantly reduced total germ cell number by 43 and 53%, respectively, compared with controls. However, germ cell proliferation was not significantly decreased (Hurtado-Gonzalez et al., 2018). These studies suggest that, in xenograft models, androgen production by human fetal testis is still sensitive to APAP, but not to IBU exposure after the male programming window, and that exposure to therapeutic doses of APAP or IBU for 7 days also targets the germline of the xenografted human fetal testes.

#### Impact on Germ Cells and Putative Intergenerational Effects

More studies have investigated the effects of analgesic exposure during pregnancy on fetal germ cell development and reproductive function in rodents. The obtained results suggest that the modifications observed in fetal germ cells might affect the reproductive potential not only of the exposed fetuses but also of future generations (intergenerational effects) (Dean et al., 2016; Rossitto et al., 2019a) (Table 2). *In vivo* administration of APAP (350 mg/kg/day between 13.5 and 21.5 dpc) or indomethacin (0.8 mg/kg/day between 15.5 and 18.5 dpc) to pregnant rats reduced the number of germ cells in fetal testes (indomethacin only) (Dean et al., 2016). *OCT4* downregulation in germ cells was significantly accelerated between 15.5 and 17.5 dpc when gonocytes enter into mitotic arrest, suggesting earlier gonocyte differentiation (Dean et al., 2016). However, the male germ cell number was compensated by early puberty and the fertility of F1 males was comparable to that of controls. F2 adult males were heavier than controls, but otherwise were normal (Dean et al., 2016).

*In utero* exposure of pregnant mice to the APAP and IBU combination (but not to each molecule alone) at low therapeutic doses (50 mg/kg/day and 15 mg/kg/day) between 10.5 and 13.5 dpc (the time of sexual determination) significantly reduced germ cell proliferation. The acceleration of germ cell differentiation (i.e., mitotic arrest) was confirmed by transcriptomic analysis of 13.5 dpc exposed testes. The male differentiation factors *Sox9* and *Dnmt3L* and extracellular matrix genes were upregulated, while factors involved in pluripotency (*Sox2*, *Lefty1*) were downregulated. Consequently, increased glycoprotein accumulation and earlier germline 5-mC deposition were detected in 14.5 dpc exposed testes, suggesting earlier somatic and germ cell differentiation (Rossitto et al., 2019a). In F1 postnatal testes, the pool of spermatogonia A was reduced and Sertoli cell maturation was delayed (Sertoli cells still expressed AMH at 30 dpp). In adult F1 animals, testosterone production was inhibited by 80% and the production of spermatozoa was significantly reduced by 20% compared with control animals, but their mobility was normal. Fertility of 3-month-old F1 animals also was normal. In adult F2 animals from parents that were both exposed to APAP + IBU, testosterone and sperm production were normal; however, sperm mobility was reduced by 40% compared with controls. At 3 months of age, F2 males were fertile, but became sub-fertile after 6 months, as indicated by the longer mating time compared with controls (> 2 weeks *vs*. 1 week). The decreased fertility might be related to impaired spermiogenesis, the final stage in sperm production that is, essential for the sperm genome compaction and reproductive capacity. Moreover, earlier replacement of histones by transition proteins and then protamines in the spermatid genome was observed (Rossitto et al., 2019a). Altogether, these results show that *in utero* exposure to APAP alone, during the sensitive period of sexual determination, affects germ cell differentiation (Dean et al., 2016; Rossitto et al., 2019a). When administrated with IBU, sperm quality of F1 animals and also of second generation F2 animals is affected, suggesting an intergenerational impact (Rossitto et al., 2019a).

#### Pubertal and Adult Exposure

Pre-pubertal exposure (23–53 dpp) to IBU (2.4–14.3 mg/kg/day) impaired sperm parameters in adult rats and compromised the next generation. The highest dose affected also sexual behavior and reduced the fertility potential in adulthood. IBU treatment reduced testosterone secretion and altered the sperm quantity and quality: decreased sperm motility and daily sperm production. In the progeny of the exposed animals, sperm transit time in the epididymis was accelerated in males, and estrous cycle was altered and fertility potential reduced in females (Barbosa et al., 2020). Moreover, in adult rats, long-term treatment with APAP at high doses (500–1,000 mg/kg for 30 days) impaired sexual competence, sperm count and quality, and fertility. These effects were the consequence of oligozoospermia, of the lack of activated sperm motility, and of increased pre-implantation defects. However, these effects were reversible (Ratnasooriya and Jayakody, 2000). Conversely, adult mouse sperm parameters were not affected by *in utero* exposure to low doses of ASA or IBU (Stutz et al., 2000).

### Female Reproductive Health

*In vivo* administration of APAP (350 mg/kg/day between 13.5 and 21.5 dpc) or indomethacin (0.8 mg/kg/day between 15.5 and 18.5 dpc) to pregnant rats reduced the number of germ cells (by 40–50%) in fetal ovaries (indomethacin only) (Dean et al., 2016). Consequently, the adult ovary size and F1 female fertility (measured by the number of pups per litter) were affected by *in utero* exposure to APAP and indomethacin (Dean et al., 2016). A delay in normal germ cell development and entry into meiosis in *in utero* exposed female rats was also observed because the pluripotency marker *Lin28* was still significantly expressed and the meiotic entry markers *Dmrt1* and *Stra8* were not completely downregulated in 18.5 dpc ovaries (Dean et al., 2016). In F2 adult females (the offspring of an indomethacin-exposed F1 parent), ovary weight was significantly decreased due to the reduction (by 60%) in primordial follicles. In agreement, serum AMH levels were significantly increased in F2 animals (Dean et al., 2016). Similarly, maternal APAP exposure (350 mg/kg/day from 6 dpc until delivery, and from 6 dpc until weaning) altered the sexual development of female offspring and resulted in impaired sexual behavior, decreased follicle reserve, and increased plasma estradiol concentrations (Aleixo et al., 2020) (Table 2).

In mice, *in utero* exposure to APAP (50 or 150 mg/kg/day from 7 dpc to delivery) also decreased the number of primordial germ cells at 13.5 dpc, leading to a reduced follicular pool in exposed ovaries compared with controls (Holm et al., 2016). Consequently, the number of full-term pregnancies and of pups per litter were decreased. This indicates that i*n utero* exposure to therapeutic doses of APAP affects female fertility (Holm et al., 2016). Furthermore, in mice, *in utero* exposure to therapeutic doses of the APAP-IBU combination during the sex determination period significantly increased the number of germ cells in 13.5 dpc female fetuses compared with controls (Rossitto et al., 2019b). Transcriptomic analysis (RNA-seq) of 13.5 dpc ovaries showed that 43 meiotic genes were downregulated (particularly pre-meiotic genes: *Stra8*, *Dazl*) and 45 meiotic genes were upregulated (genes involved later in meiosis: *Sycp1*, *Rec8*, *Dmc1*) in *in utero* exposed gonads compared with controls. This confirms that upon exposure to the APAP and IBU combination, the meiotic program is deregulated in mouse oocytes. Consequently, in postnatal F1 (exposed) and F2 (offspring of exposed F1 parents) animals, the primordial follicle pool was increased and oocyte activation (increased oocyte diameter) was accelerated compared with controls. This phenotype was accompanied by an increase in apoptosis of growing follicles at 14 and 21 dpp. At the molecular level, the *KitL*, *FshR*, *Bmp15*, *Gdf9* genes that contribute to oocyte activation were significantly upregulated in F1 ovaries, whereas the PI3K-AKT-FOXO3 signaling pathway (downstream of these genes) was activated in 8 dpp F1 (directly *in utero* exposed) and F2 ovaries, suggesting that APAP-IBU exposure might interfere with the transition from primordial to primary follicles (Rossitto et al., 2019b). Fertility of 2-month-old F1 and F2 females was normal; however, 6-month-old F2 females were sub-fertile as indicated by the smaller litter size, although the mating time was similar to controls. The follicular stock in F1 and F2 ovaries started to decrease (AMH expression quantification) already at 2 months post-partum. Furthermore, in 6-month-old F1 ovaries, the number of corpora lutea was increased (up to 30 per ovary) due to impaired regression following their increased cell proliferation and decreased apoptosis (Rossitto et al., 2019b). This phenotype is similar to the premature ovarian insufficiency phenotype described in women (Huhtaniemi et al., 2018).

Altogether, these *in vivo* experiments in rodent models show that exposure to NSAIDs and/or mild analgesics during the early gonad development period can interfere with somatic cell differentiation and with germline proliferation/differentiation in the male and female gonads (Table 2). Furthermore, intergenerational effects to the non-exposed offspring have been identified, leading to decreased fertility in F2 (i.e., not directly exposed) animals.

## Conclusion

Globally, these *in vitro*/*ex vivo* studies using organotypic culture of human and rat gonad explants and xenografted mice and the *in vivo* experiments in rodents strongly suggest a link between prenatal or adult exposure to human equivalent therapeutic doses of NSAIDs/analgesics and negative effects on the reproductive tract development and function. In agreement with the findings of many epidemiological studies, these experimental studies identified analgesics and NSAIDs as endocrine disruptors. However, results from experimental studies indicate that the anti-androgenic activity of NSAIDs/analgesics is only partially coupled to inhibition of PG synthesis, and that other mechanisms, unrelated to COX inhibition, are probably implicated in the endocrine regulation in response to these drugs (e.g., LOX, CYP450 or endocannabinoid pathways). Additional studies are needed to elucidate whether other AA metabolites are involved in the effects induced by exposure to NSAIDs, and whether other NSAID targets play a role in their hormonal effects in gonads. Also, the effects of exposure to environmental doses of APAP, IBU, or DCF (10–50 ng/ml in drinking water, corresponding to an exposure of a few ng/kg/day in humans or rodents) still need to be determined.

*In vivo* studies suggest that the time windows corresponding to gonadal sex determination, embryonic germ cell differentiation, mitotic arrest in males and meiosis entry in females are particularly sensitive to the effects of these drugs. Recent data in the rat and mouse explained how fetal exposure to these drugs is associated with earlier and delayed fetal germ cell differentiation in males and females, respectively. Transcriptomic analyses allowed the identification of molecular targets of these drugs in the somatic and germ cell lineages. Furthermore, abnormal testis and ovary development and function in the F2 generation suggests an intergenerational effect *via* both parents that may be a consequence of epigenetic changes in germ cells. More experiments are needed to determine the epigenetic regulations of these processes.

Exposure to these pharmaceutical molecules affects the production of fetal sexual hormones, thus promoting the development of male and female reproductive disorders that lead to impaired fertility or shorter reproductive lifespan in adult animals (Kristensen et al., 2016; Hurtado-Gonzalez and Mitchell, 2017; Kristensen et al., 2018; Kilcoyne and Mitchell, 2019). These findings in animal models indicate that exposure to NSAIDs/analgesics cause reproductive side effects that are of concern also for humans. Recently, it has been recommended to use APAP with caution during pregnancy on the basis of the many data on the adverse effects on neurological and reproductive health associated with the maternal and perinatal use of APAP (Bauer et al., 2021). As people often take analgesics that contain also one NSAID or are exposed to various cocktails of anti-androgenic or anti-estrogenic molecules in the environment, studies using combination of analgesics should be generalized. Although fetal gonad development and reproductive tract formation and function are not fully overlapping between rodents and humans (Habert et al., 2014; Sharpe, 2020), more studies in rodents should be performed to assess the negative effects of these molecules, due to the high prevalence of analgesic use in pregnancy and the uncontrolled presence of these molecules in the environment.
